# Comparison of Histochemical Stainings in Evaluation of Liver Fibrosis and Correlation with Transient Elastography in Chronic Hepatitis

**DOI:** 10.1155/2015/431750

**Published:** 2015-11-17

**Authors:** Daniela Cabibi, Fabrizio Bronte, Rossana Porcasi, Sabrina Ingrao, Antonino Giulio Giannone, Marcello Maida, Maria Grazia Bavetta, Salvatore Petta, Vito Di Marco, Vincenza Calvaruso

**Affiliations:** ^1^Department of Sciences for the Promotion of Health, Section of Pathology, University of Palermo, Palermo, Italy; ^2^Gastroenterology and Hepatology Unit, Di.Bi.M.I.S., University of Palermo, Palermo, Italy

## Abstract

*Background and Aim*. The best staining to evaluate liver fibrosis in liver hepatitis is still a debated topic. This study aimed to compare Masson's trichrome (MT), Sirius Red (SR), and orcein stainings in evaluating liver fibrosis in chronic HCV hepatitis (CHC) with semiquantitative and quantitative methods (Collagen Proportionate Area (CPA) by Digital Image Analysis (DIA)) and correlate them with transient elastography (TE). *Methods*. Liver stiffness evaluation of 111 consecutive patients with CHC was performed by TE. Semiquantitative staging by Metavir score system and CPA by DIA were assessed on liver biopsy stained with MT, SR, and orcein. *Results*. MT, SR, and orcein staining showed concordant results in 89.6% of cases in staging CHC, without significant difference in both semiquantitative and quantitative evaluations of fibrosis. TE values were concordant with orcein levels in 86.5% of the cases and with MT/RS in 77.5% (*P* < 0.001). No significant correlation between the grade of necroinflammatory activity and TE values was found. *Conclusion*. In CHC, SR/MT and orcein stainings are almost concordant and when discordant, orcein staining is better related to TE values than MT/RS. This suggests that elastic fibers play a more important role than reticular or collagenous ones in determining stiffness values in CHC.

## 1. Introduction 

Liver biopsy is considered the gold standard for assessing the presence of fibrosis in viral hepatitis and many score systems and stainings have been proposed to stage liver biopsies. Pathologists usually perform more than one staining to assess fibrosis in liver diseases, such as silver impregnation for reticulin fibers, chromotrope-aniline blue for collagen, Masson trichrome (MT), Sirius red (SR), Victoria blue, and orcein staining for elastic fibers.

The results of these stainings are not always overlapping because they highlight the different connective components increasing during the progression of the disease. SR and MT stainings highlight the collagenous component of liver fibrosis, whereas, according to Scheuer and Lefkowtich [1], Victoria blue or orcein staining is important to highlight the elastic fibers, usually absent in normal portal tracts.

SR is the preferred staining to best quantify the liver fibrosis by recently introduced techniques, such as the computer-assisted Digital Image Analysis (DIA) of liver collagen [2, 3].

Previous studies showed that the amount of liver collagen obtained with DIA is significantly correlated with semiquantitative evaluation of liver fibrosis, Hepatic Venous Pressure Gradient (HVPG), and clinical decompensation in HCV-infected post-LT patients [4, 5] suggesting a prognostic role of this tool in this setting.

Moreover, transient elastography (TE), a recent noninvasive method to assess hepatic fibrosis by FibroScan (Echosens) [6], has spread widely in the clinical management of liver diseases. Studies comparing which of the above-mentioned histochemical stainings for liver fibrosis better correlate with TE results on chronic hepatitis cases are lacking.

The aim of this study is to compare semiquantitatively and quantitatively the different histological stainings to assess fibrosis and verify the correlation with the presence of necroinflammatory activity and with liver stiffness measurement by TE in chronic HCV hepatitis cases (CHC).

## 2. Materials and Methods

The study was prospectively performed on a total of 111 consecutive samples of liver biopsies of patients evaluated at the Pathology Department of the University Hospital of Palermo (70 males and 41 females, age between 16 and 76 years), collected between January 2011 and December 2014.

They consisted of 111 cases of CHC.

In order to reliably evaluate grade and stage of the liver damage, biopsies with length at least of 15 mm were included in the studies, as previously recommended [7].

The slides were stained with Hematoxylin and Eosin (H&E), Shikata's orcein, and MT and SR stainings. The stains were made on seriated sections of the same level for every case.The previously reported protocols were applied for these stainings [1, 8].

SR staining is obtained by dissolving 0.5 g of Sirius Red in 500 mL of saturated aqueous solution of picric acid. It is specific for many types of collagen (types I, II, III, IV, and V). We do not counterstain the nuclei with hematoxylin because the addition of another color could make it difficult to perform DIA. In this way, SR is useful to quantify the total collagen in liver biopsies because it produces a dual color staining, with collagen fibers stained in red and parenchyma in light pink.

Semiquantitative assessment of grading and staging of CHC cases was performed according to Metavir score system [9, 10]. Grading was assessed on HE slides and staging was separately assessed on Shikata's orcein and SR and MT stained slides by a liver pathologist (DC), by interpreting the series without knowledge of the Metavir stage obtained with each staining. For evidencing elastic fibers, we performed orcein staining and not Victoria blue because orcein like SR provides a better staining for DIA than trichrome stainings [2, 3].

DIA was performed on 50 chronic hepatitis cases by using the Zeiss AxioVision Rel. 4.8 image analysis software on digitalized images of Shikata's orcein and SR stained sections. DIA has been acquired by scanning the entire slide through histology slide scanner (Aperio ScanScope) at resolution 1x. DIA was performed by 1 author (VC) blinded to clinical, laboratory, and histological information.

**Figure 1:**
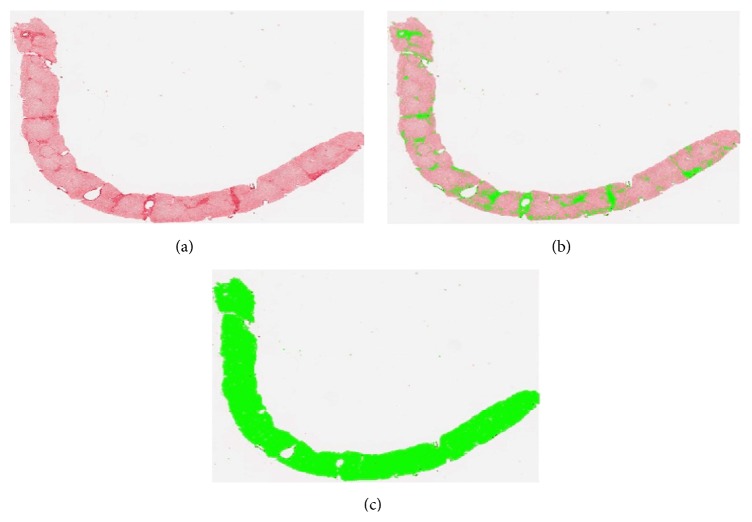
(a) Digitized image of the entire scanned section (Sirius red staining). (b) Selection of the Sirius red stained fibrous areas. (c) Selection of the area of the entire scanned section.

**Figure 2:**
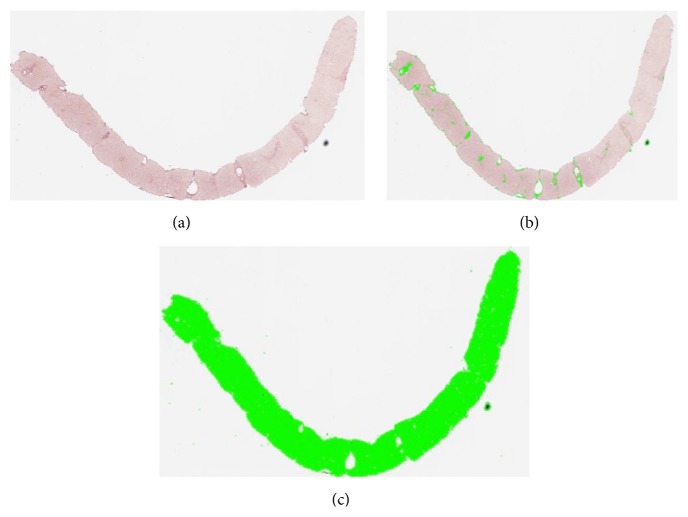
(a) Digitized image of the entire scanned section (orcein staining). (b) Selection of the orcein stained fibrous areas. (c) Selection of the area of the entire scanned section.

The quantitative analysis involves the following steps (Figures 1 and 2):

- acquisition of the image by AxioVision software;
- processing and quantitative measurement of the stained fibrous areas using a specific protocol of AxioVision software for the quantitative analysis with an automated thresholding used for all the slides of each staining;
- percentage ratio between the fibrotic area and the entire sample.

The statistical analysis of the results was performed with Student's* t*-test of the package SPSS 15.0. Continuous variables were expressed as mean ± standard deviation (SD) and categorical variables as absolute and relative frequencies. The differences between continuous data were analyzed by* t*-test, and corrected *χ* ^2^ analysis was used for dichotomous or categorical variables. The results were considered statistically significant at *P* < 0.05.

The liver stiffness measurement was assessed on the whole casuistry of 111 cases at the time of biopsy, through FibroScan (Echosens, Paris).

Liver stiffness (LS) was evaluated in the right lobe of the liver through intercostal spaces, with the patient in the supine position and the right arm in maximal abduction. In all patients, at least 10 measurements of LS were taken. The success rate was calculated as the ratio of the number of successful measurements to the total number of acquisitions. Median values of the successful measurements were kept as representative of LS, and results were expressed in kilopascal (kPa). Only examinations with at least 10 valid measurements and a success rate of more than 60% were considered reliable.

TE of the liver was performed by one expert physician (FB) who is proficient at the use of fibroelastometry in patients with chronic liver disease (more than 3000 independent procedures performed).

The reference scheme that we used to correlate the values of the liver stiffness and the histological stage of fibrosis was the following [11]:

 stages 0-1: <7.1 kilopascals (kPa);
 stage 2: ≥7.1 kPa;
 stage 3: ≥9.5 kPa;
 stage 4: ≥12.5 kPa.

In keeping with this scheme, the cut-off value of 9.5 kPa has been used to divide chronic hepatitis cases in two groups and to verify their concordance with the histological fibrosis stage (stages 1-2 versus stages 3-4).

## 3. Results

MT and SR staining showed overall the same results in staging CHC (percentage of concordance 95%). So, we will report together the results of the two stainings as “Masson trichrome/picrosirius staining (MT/SR).”

**Figure 3:**
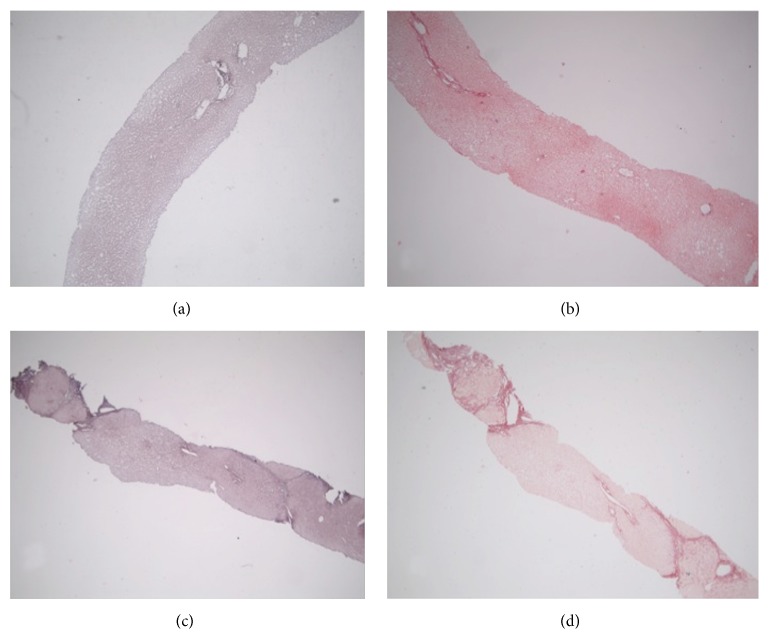
(a, b) Group A: orcein (a) and picrosirius (b) concordant, stage 1/2. (c, d) Group B: orcein (a) and picrosirius (b) concordant, stage 3/4. Original magnification: (a, b, c, d) 4x.

**Figure 4:**
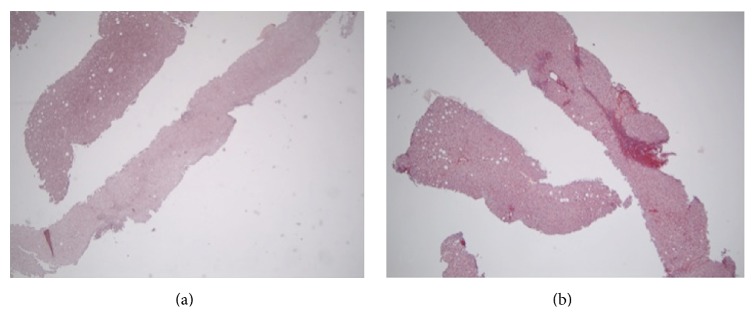
(Group C case): (a) orcein staining showing only mild portal fibrosis (stage 1). (b) Picrosirius staining showing some collagenous bridges (stage 3). Original magnification: (a-b) 4x.

CHC cases consisted of 25, 42, and 44 cases with, respectively, mild (A1), moderate (A2), and severe (A3) grade of necroinflammatory activity. They were grouped into three groups, based on the concordance in histological staging based on MT/SR and orcein slides:

 Group A: 73 cases with orcein and MT/SR concordant stages 1-2 (Figures 3(a) and 3(b)).
 Group B: 26 cases with orcein and MT/SR concordant stages 3-4 (Figures 3(c) and 3(d)).
 Group C: 12 cases with orcein and MT/SR discordant results (orcein stages 1-2 versus MT/SR stages 3-4; no cases showed the reverse) (Figures 1, 2, 4(a), and 4(b)).

So, in CHC, the three stainings gave concordant results in 99/111 cases (89.6%, i.e., groups A and B cases), while only in 12/111 (10.4%, i.e., group C cases) the results of the stainings were discordant. By statistical comparison, no significant difference was found among the three stainings in staging liver fibrosis (*P* > 0.05).

Among the 50 patients analyzed by DIA, mean CPA value was 4.9 ± 4.5%, 4.2 ± 2.6%, 6.9 ± 1.2%, and 15.4 ± 7.0% on SR stained sections and 3.7 ± 1.5%, 4.7 ± 3.9%, 6.0 ± 1.4%, and 16.9 ± 7.2% on Shikata's orcein stained sections, respectively, for stages I, II, III, and IV.

When statistical analysis was performed stage by stage on the above-reported DIA results, no statistically significant difference was observed between mean CPA value of SR and orcein stains (*P* > 0.05 for all stages), suggesting that, in CHC, SR and orcein staining give overlapping results (Table 1).

## 4. Correlation with TE

The 111 cases of CHC were grouped in Table 2, according to the histological staging and TE values.

In group A, 64/73 cases (87.7%) showed TE values < 9.5 kilopascals and 9 showed TE values > 9.5 kilopascals (*P* < 0.001).

In group B, 21/26 (80%) showed TE values > 9.5 kilopascals and 5/26 showed TE values < 9.5 kilopascals (*P* = 0.002).

So, 85/111 CHC cases (77%) showed concordant results for MT/RS, orcein, and TE values.

Group C consisted of cases with discordant results (orcein stages 1-2 versus MT/RS stages 3-4). 11/12 showed TE values < 9.5 kilopascals, in keeping with the low orcein results, and only 1/12 showed TE values > 9.5 kilopascals (*P* = 0.007), Table 3. No case showed cholestasis or siderosis. Steatosis was absent in 5/12 cases and mild (<33%) in 6/12 cases. Only 1/12 cases showed moderate steatosis (40%). No patients had BMI higher than 30 kg/m^2^.

So, overall, 96/111 cases (86.5%) were concordant for orcein and TE values, while only 86/111 cases (77.5%) showed concordance between MT/RS and TE values (*P* < 0.001).

Finally, in group C cases and in group A cases, showing discordant values between histological MT/SR/orcein fibrosis and TE values, no significant correlation between the grade of necroinflammatory activity and TE values was found (*P* > 0.05).

## 5. Discussion

Routine fibrosis assessment is usually carried out on trichrome or reticulin staining, respectively, evidencing collagenous and reticular fibers [3]. It is noteworthy that, according to Scheuer and Lefkowtich [1], Victoria blue and orcein stainings highlight elastic fibers, usually absent in normal portal tracts. The importance of the histochemical stainings for elastic fibers has been stressed to distinguish old fibrosis of the advanced stages of chronic hepatitis, in which elastic fibers are the product of active formation of true septa, from collapse of the reticular network that, for example, in acute hepatitis, is subsequent to recent parenchymal necrosis, with the formation of collagenous bridges that could lead to a misdiagnosis of cirrhosis if collagenous and/or reticulin stainings are used to assess the fibrosis [1, 12].

Recently, we performed a comparative semiquantitative and quantitative study on acute hepatitis cases [13]. We observed a high rate of discordance between MT/RS and orcein staining, with MT/RS showing advanced fibrosis (stages III-IV), quantitatively confirmed by high values of CPA and related to high stiffness values, but not supported by the paired orcein stained slides, showing absent or mild fibrosis and low values of CPA on orcein stained sections. Thus, to achieve the diagnosis of acute hepatitis, by definition lacking of significant elastic fibrosis, orcein seems to perform better than TE and MT/RS, the latter potentially suggesting a misdiagnosis of severe fibrosis.

In the present study, instead, 89% of CHC cases showed MT/RS and orcein concordant results, well correlating with TE values.

The study has limitations. The semiquantitative evaluation of fibrosis has been performed by a single operator; thus, the interobserver concordance has not been assessed. However, for about half of the cases in which DIA was performed, the quantitative results were strictly concordant with the semiquantitative results of Metavir score system (CPA value was 4.9 ± 4.5%, 4.2 ± 2.6%, 6.9 ± 1.2%, and 15.4 ± 7.0% on MT/SR stained sections and 3.7 ± 1.5%, 4.7 ± 3.9%, 6.0 ± 1.4%, and 16.9 ± 7.2% on Shikata's orcein stained sections, resp., for the semiquantitatively assessed stages I, II, III, and IV). So, in our opinion, DIA can be used as a control of the reliability of the semiquantitative assessment of fibrosis by the observer.

It is noteworthy that TE values were better related to orcein stage (86.5%) than to MT/SR stage (77.5%). This was well evident in group C cases in which 11/12 cases showed orcein stages 1-2, MT/SR stages 3-4, and stiffness value < 9.5 kPa. Moreover, no statistically significant correlation was found between TE values and the severity of the necroinflammatory activity, nor with the presence of steatosis.

Furthermore, among the 12 patients in group C, only one showed moderate steatosis, and no one had BMI value higher than 30 kg/m^2^ [14] nor showed cholestasis or siderosis. Furthermore, TE was performed by one senior operator to reduce the interobserver variability [15, 16]. Thus, we have not found any clinical factors which can reduce the reliability of TE in our patients.

Previous studies reported that, in acute hepatitis or in chronic hepatitis of viral origin with severe necroinflammation, the liver stiffness values are greatly increased and correlate with high values of aminotransferases [17–19]. We agree with these observations concerning acute hepatitis cases, where we previously evidenced a strong correlation between TE values and MT/SR, but not with orcein levels [13]. On the contrary, in CHC, we were not able to find this correlation because orcein and MT/RS gave almost concordant results, with TE values showing better correlation with orcein than with MT/RS when the stainings gave discordant results. So, we can hypothesize that, different from acute hepatitis, in CHC, in which the acute inflammation usually plays a less prominent role, the long term inflammation leads to the real increase of the elastic fibers, better evidenced by orcein staining and well correlating with TE values. Overall, MT/RS and orcein stainings and TE showed sufficiently concordant results in CHC, independently of the severity of necroinflammatory activity and steatosis. Nevertheless, this is not true for the 12 patients of group C staged F1-2 instead of F3-4, in which orcein staining and TE appear suitable for fibrosis evaluation, more than MT/RS. Thus, as for acute hepatitis [13], we recommend always to perform elastic fibers stains together with SR and to be very cautious when they give discordant results. In these cases, to better evaluate the fibrosis staging, the clinical data and TE are very useful and in our experience they better correlate with orcein results. In our opinion, the elastic fiber stains are not really less sensitive in detecting the fibrous tissue, but they seem so when they are erroneously compared with MT/SR that highlight a recent, still reversible, collagenous increase as previously demonstrated in acute hepatitis [13]. To our knowledge, this is the first study in which DIA was comparatively assessed on picrosirius and orcein stained sections of CHC cases and then correlated with TE results.

## Figures and Tables

**Table 1:** Comparison of DIA results obtained on Masson trichrome/Sirius Red and orcein stained slides. The comparison has been performed separately for each stage.

	MT/SR (%) ± DS	Orcein staining (%) ± DS	*P* value
Chronic hepatitis (50 pts)			
Stage 1 (12 pts)	4.9 ± 4.5	3.7 ± 1.5	0.126
Stage 2 (10 pts)	4.2 ± 2.6	4.7 ± 3.9	0.808
Stage 3 (8 pts)	6.9 ± 1.2	6.0 ± 1.4	0.234
Stage 4 (20 pts)	15.4 ± 7.0	16.9 ± 7.2	0.467

**Table 2:** FibroScan values and histological stages in 111 patients with CHC. Group A: cases with Masson's trichrome/Sirius red and orcein concordant stages 1-2. Group B: cases with Masson's trichrome/Sirius red and orcein concordant stages 3-4. Group C: cases with Masson's trichrome/Sirius red and orcein discordant results (orcein stages 1-2 versus Masson's trichrome/Sirius red stages 3-4; no cases showed the reverse).

	Stiffness < 9.5	Stiffness > 9.5	*P* value
Number of cases = 111	**80**	**31**	
Group A (stages I-II) = 73 cases	64	9	<**0.001**
Group B (stages III-IV) = 26 cases	5	21	**0.002**
Group C = 12 cases			
*Orcein* = stages 1-2; MT/SR = stages 3-4	11	1	**0.007**
*Orcein* = stages 3-4; MT/SR = stages 1-2	0	0	
	0	0	

**Table 3:** Clinical and histological features of group C patients.

	Age	Platelets (*∗*10^3^)	Ast/Alt (U/L)	BMI (kg/m^2^)	Steatosis (%)	Grade	SR stage	Orcein stage	CPA SR	CPA orcein	TE (kPa)
1	25	224	221/578	24	0	2	2	1	5.8	2.4	6.1
2	51	209	31/27	26	0	2	3	2	8.5	5.3	6.4
3	37	221	76/98	27	0	3	3	1	8.2	3.7	8.7
4	61	172	29/28	23	0	1	3	1	7.9	4.1	6.3
5	51	200	153/257	27	20	3	3	2	8.4	5.8	7.9
6	52	203	70/104	24	20	3	3	1	6.2	3.5	6
7	56	341	19/14	28	10	3	3	2	7.8	4.5	4.8
8	59	155	24/23	22	0	3	4	2	15.4	4.5	5.6
9	30	226	25/29	24	40	2	3	2	7.7	4.6	6.3
10	43	197	46/89	28	10	3	3	2	7.9	3.8	6.0
11	57	135	292/294	24	0	3	3	1	10.6	6.3	13.8
12	69	219	53/75	27	10	3	3	2	6.7	3.2	5.6
